# The Heterologous Expression of the p22 RNA Silencing Suppressor of the Crinivirus Tomato Chlorosis Virus from Tobacco Rattle Virus and Potato Virus X Enhances Disease Severity but Does Not Complement Suppressor-Defective Mutant Viruses

**DOI:** 10.3390/v9120358

**Published:** 2017-11-24

**Authors:** Yazmín Landeo-Ríos, Jesús Navas-Castillo, Enrique Moriones, M. Carmen Cañizares

**Affiliations:** Instituto de Hortofruticultura Subtropical y Mediterránea “La Mayora”—Universidad de Málaga—Consejo Superior de Investigaciones Científicas (IHSM-UMA-CSIC), Estación Experimental “La Mayora”, Algarrobo-Costa, 29750 Málaga, Spain; landeoyazmin@gmail.com (Y.L.-R.); jnavas@eelm.csic.es (J.N.-C.); moriones@eelm.csic.es (E.M.)

**Keywords:** *Closteroviridae*, *Crinivirus*, *Tomato chlorosis virus*, RNA silencing suppressor, virus pathogenicity

## Abstract

To counteract host antiviral RNA silencing, plant viruses express suppressor proteins that function as pathogenicity enhancers. The genome of the Tomato chlorosis virus (ToCV) (genus *Crinivirus*, family *Closteroviridae*) encodes an RNA silencing suppressor, the protein p22, that has been described as having one of the longest lasting local suppressor activities when assayed in *Nicotiana benthamiana*. Since suppression of RNA silencing and the ability to enhance disease severity are closely associated, we analyzed the effect of expressing p22 in heterologous viral contexts. Thus, we studied the effect of the expression of ToCV p22 from viral vectors Tobacco rattle virus (TRV) and Potato virus X (PVX), and from attenuated suppressor mutants in *N. benthamiana* plants. Our results show that although an exacerbation of disease symptoms leading to plant death was observed in the heterologous expression of ToCV p22 from both viruses, only in the case of TRV did increased viral accumulation occur. The heterologous expression of ToCV p22 could not complement suppressor-defective mutant viruses.

## 1. Introduction

RNA silencing acts as an effective antiviral defense in plants [1]. To counteract it, plant viruses have developed strategies based on the expression of silencing suppressor proteins. Most of the viral RNA silencing suppressors identified so far also enhance virus accumulation and pathogenicity [2,3]. These suppressors of RNA silencing may contribute to disease synergism in which one virus enhances the virulence or complements the defects of the other one by helping it to replicate or to move systemically. In viral synergism, co-infection of two or more viruses can exacerbate symptom severity and increase the titer of the viruses observed in single infections [3,4]. The best studied example of plant viral synergism is that between Potato virus Y (PVY, genus *Potyvirus*, family *Potyviridae*) and Potato virus X (PVX, genus *Potexvirus*, family *Alphaflexiviridae*) in tobacco [4,5,6,7,8]. The pathogenicity enhancement observed when the potyviral helper component-proteinase (HC-Pro) suppressor was expressed from heterologous viruses revealed that this was the sequence involved in the synergistic interaction [3]. Similarly, the co-expression of other viral RNA silencing suppressors enhances PVX virulence [9,10].

Tomato chlorosis virus (ToCV, genus *Crinivirus*) belongs to the family *Closteroviridae*, in which the largest RNA genomes among plant viruses have been reported [11]. ToCV is transmitted in nature by the whitefly (Hemiptera: Aleyrodidae) *Bemisia tabaci* and has a bipartite single-stranded, positive-sense RNA genome [12,13,14,15]. As for other members of the family *Closteroviridae*, ToCV adopts the strategy of encoding multiple RNA silencing suppressors [16]. Thus, while ToCV RNA-2 has delegated its suppressor function to the structural proteins coat protein (CP) and minor coat protein (CPm), RNA-1 encodes an apparently dedicated suppressor protein, p22, although no studies have been performed to know if it is implicated in other steps of the viral infection process. Interestingly, p22 has been shown to be one of the longest lasting local suppressors reported to date when assayed in *Nicotiana benthamiana*, suppressing local RNA silencing induced either by sense RNA or dsRNAs [16] and binding preferentially long dsRNAs [17]. Moreover, previous work showed that the heterologous expression of p22 from PVX led to plant death [16].

Tobacco rattle virus (TRV, genus *Tobravirus*, family *Virgaviridae*) and PVX have been frequently used as viral vectors for both, gene expression and gene silencing studies [18,19,20]. TRV has a bipartite, positive-sense single-stranded RNA in which RNA1 encodes the replicase proteins, the 29K cell-to-cell movement protein [21], recently described as an RNA silencing suppressor [22], and the 16K silencing suppressor protein [23,24,25,26]. TRV RNA2 encodes a coat protein (CP) [27], and it has been engineered to facilitate insertion of heterologous gene sequences [28,29,30,31]. In RNA1, while the replicase is translated from the genomic RNA, the 29K and 16K proteins are translated from the respective subgenomic RNAs (sgRNA) [27]. TRV RNA1 is capable of replicating and moving systemically in plants in the absence of RNA2 [32]. On the other hand, PVX has a monopartite, positive-sense single-stranded RNA genome encoding five open reading frames (ORFs); a first ORF encodes the viral replicase, a central region encodes three overlapping ORFs, known as the triple-gene block (TGB), and a final ORF encodes the CP [33]. It has also been engineered to insert heterologous gene sequences. The three central region-encoded proteins and the final viral CP are translated from sgRNAs. One of the three TGB proteins is the multifunctional P25 that suppresses RNA silencing and is required for cell to cell movement through plasmodesmata [34,35]. It has been shown that although PVX movement is dependent on the suppression function of P25, suppression of silencing is not sufficient to allow virus movement [35].

In this study, we assayed the role of p22 viral silencing suppressor as enhancer of disease severity in heterologous viral infections using as host plant *N. benthamiana*. Both wild-type and attenuated TRV and PVX suppressor mutants were examined for heterologous expression of p22. Our results show that, although p22 induced a dramatic enhancement of the disease symptoms caused by TRV and PVX, resulting in plant death, the mechanism involved in this process seemed to be different for each virus. Thus, while the increase in TRV pathogenicity is linked to an increased viral RNA level in plants, no change was observed for PVX. Also, the heterologous expression of ToCV p22 did not functionally complement the absence of the 16K or P25 suppressors of defective TRV or PVX mutant viruses, respectively, at both the local and systemic level.

## 2. Materials and Methods

### 2.1. Plasmid Constructs

Plasmids pTRV1 and pTRV2 containing the cDNA of TRV RNA1 and RNA2, respectively, under the Cauliflower mosaic virus (CaMV) 35S promoter have been described [29]. To construct a TRV expressing ToCV p22 (TRV2p22), the cDNA fragment corresponding to ToCV p22 was PCR amplified using the Expand High Fidelity PCR system (Roche Diagnostics, Mannheim, Germany) and primers MA 1287 and MA 1288 (Supplementary Materials, Table S1) with specific restriction sites and cloned into pTRV2. For mutants TRV1∆16K and TRVΔ16Kp22, one premature stop codon was introduced at position 11 of the amino acid sequence of the 16K ORF by PCR using the QuikChange II XL Site-Directed Mutagenesis Kit (Stratagene, Agilent Technologies, Santa Clara, CA, USA) and specific primers MA 1281 and MA 1282 (Supplementary Materials, Table S1). Each construct was verified by DNA sequencing with an ABI 3730 XL DNA analyzer (Applied Biosystems, Foster City, CA, USA) (Macrogen Inc., Seoul, Korea). The constructs were transformed into *Agrobacterium tumefaciens* strain GV3101 by electroporation.

Plasmid pgR107 containing the cDNA of PVX (pPVX) under the CaMV 35S promoter has been described [36]. The generation of a PVX expressing ToCV p22 (construct PVXp22) has also been described [16]. To generate PVX∆P25 and PVX∆P25p22 constructs, two premature stop codons were introduced at positions 23 and 25 of the amino acid sequence of the P25 ORF of the vectors PVX and PVXp22 by PCR as described above, using the specific primers MA 1621 and MA 1622 (Supplementary Materials, Table S1). Each construct was verified by DNA sequencing with an ABI 3730 XL DNA analyzer (Applied Biosystems, Foster City, CA, USA) (Macrogen Inc., Seoul, Korea). pgR107 was kindly provided by Dr. David C. Baulcombe (Sainsbury Laboratory, Norwich, UK). The constructs were transformed into *A. tumefaciens* strain GV3101 by electroporation.

### 2.2. Plant Material and Agroinfiltration

*N. benthamiana* plants (3–5 leaf growth stage) were agroinfiltrated with *A. tumefaciens* strain GV3101 carrying pTRV1 + pTRV2p22 or pTRV2, pTRV1Δ16K + pTRV2p22 or pTRV2, and pPVXp22, pPVX, pPVXΔP25p22, or pPVXΔP25. For co-infiltration of pTRV1 and pTRV2 and its derivatives, equal volumes of individual *A. tumefaciens* cultures (optical density at 600 nm of 1) were mixed prior to infiltration. Plants were maintained in a controlled temperature chamber at 25 °C with a 16 h/8 h light/dark cycle.

### 2.3. RNA Analysis

For northern blot analysis, total RNA was extracted from leaf tissues as described previously [37]. Total RNA (5 µg) was separated on 1% formaldehyde agarose gels, transferred to positively-charged nylon membranes (Roche Diagnostics, Mannheim, Germany), and probed with digoxigenin-labeled specific RNA probes for the RNA1 and RNA2 3′-untranslated region of TRV (6548–6789 nt of RNA1 and 1860–2101 nt of RNA2), and for the 3′-end region of PVX (5875–6403 nt of PVX cDNA sequence in pgR107 vector). For tissue blot hybridization, fresh cross-sections of four non-inoculated leaf petioles or stems were squash blotted on positively-charged nylon membranes (Roche Diagnostics, Mannheim, Germany), and blots were hybridized with the digoxigenin-labeled RNA probes mentioned above. The hybridization conditions used were those recommended by the manufacturer (DIG Application Manual for Filter Hybridization, Roche Diagnostics, Mannheim, Germany). Membranes were exposed to X-ray film (X-Omat AR, Kodak, Rochester, NY, USA) and developed following a conventional photographic process.

## 3. Results

### 3.1. Effect of the Heterologous Expression of the ToCV p22 Suppressor on TRV Virulence

**Figure 1:**
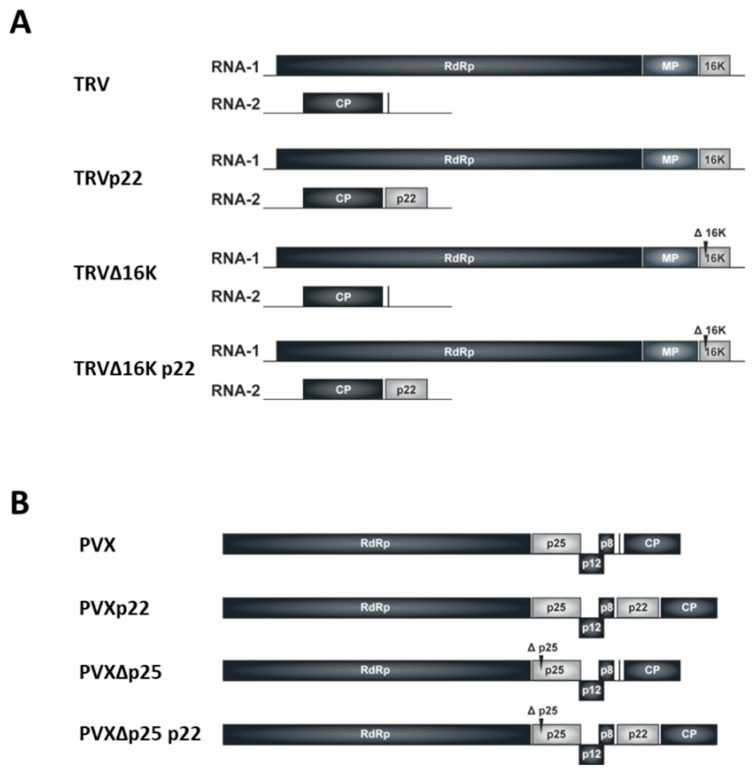
Schematic representation of Tobacco rattle virus (TRV) and Potato virus X (PVX) constructs used in this work. (**A**) TRV is the viral vector containing the infectious TRV cDNA. In TRVp22 the Tomato chlorosis virus (ToCV) p22 gene was inserted into a multiple cloning site (Mcs) on the viral RNA2 created after removing the two genes involved in nematode transmission of TRV. TRV∆16K consists of an infectious TRV cDNA that harbors a premature stop codon in the RNA1-encoded 16K open reading frame (ORF) (arrowhead). In TRV∆16Kp22, the ToCV p22 gene was inserted into the Mcs and a premature stop codon was created in the RNA1 encoded 16K ORF (arrowhead); (**B**) PVX is the viral vector containing the infectious PVX cDNA. PVXp22 consists of an infectious PVX cDNA clone that expresses ToCV p22 from a duplicated PVX CP promoter. PVX∆P25 consists of an infectious PVX cDNA clone harboring two stop codons in the P25 ORF (arrowhead). PVX∆P25p22 consists of an infectious PVX cDNA clone that expresses ToCV p22 and harbors two stop codons in the P25 ORF (arrowhead). A vertical black line shows the multiple cloning site.

To investigate the role of the ToCV p22 suppressor in the context of a heterologous TRV infection, we examined the effect of its expression from the heterologous wild-type TRV virus (TRVp22 construct), and from a 16K suppressor deficient TRV mutant (TRV∆16Kp22 construct), comparing the results with those obtained with the wild type TRV and a 16K suppressor deficient TRV mutant (TRV and TRV∆16K constructs, respectively). *N. benthamiana* plants were co-infiltrated with *A. tumefaciens* strains harboring a mixture of TRV1 and TRV2p22 or TRV2, and TRV1∆16K plus TRV2p22 or TRV2 (Figure 1A).

**Figure 2:**
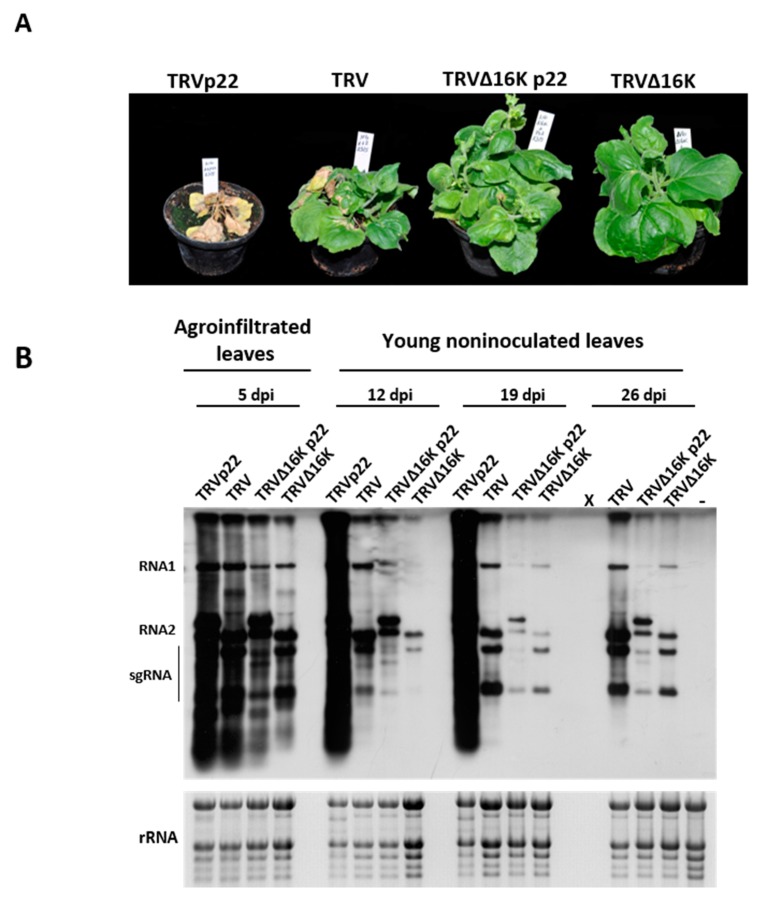
Analysis of *Nicotiana benthamiana* plants inoculated with Tobacco rattle virus (TRV) expressing Tomato chlorosis virus p22 (TRVp22), TRV, TRV∆16Kp22, and TRV∆16K. (**A**) Symptoms on *N. benthamiana* following inoculation with TRVp22, TRV, TRV∆16Kp22, and TRV∆16K at 19 days post-inoculation; (**B**) Northern blot analysis of RNA extracted from agroinfiltrated patches at five days post-infiltration (dpi) and from non-inoculated leaves at 12, 19, and 26 dpi of the same plants. Hybridization was carried out with a probe specific to the 3′untranslatable region that is identical in RNA1 and RNA2 of TRV. Positions of TRV genomic RNA1 and RNA2 and subgenomic RNAs (sgRNA) are indicated. Note that RNA2 was longer in TRVp22 and TRV∆16Kp22 because of the insertion of the p22 gene sequence. X indicates no RNA sample since plants agroinfiltrated with TRVp22 were dead at 26 dpi, and—is a healthy negative control. Ethidium bromide staining of rRNA was used as a loading control.

By 7–9 days post inoculation (dpi), a striking enhancement of symptoms was only observed in plants infected with TRVp22. TRVp22-infected *N. benthamiana* plants developed large necrotic lesions on the stems, leaf petioles, and young leaves; these spread rapidly to the whole plant, resulting in plant death by 19–21 dpi (Figure 2A). In contrast, no such severe symptoms were observed in the other treatments in which the plants exhibited symptoms similar to those of the wild type TRV infection—i.e., leaf distortion, chlorotic mottle, and mild necrosis (Figure 2A)—although they were less severe for TRV∆16K and TRV∆16Kp22. The results obtained with TRV∆16Kp22 show that no symptom enhancement occurred when both ToCV p22 and the described TRV 29K suppressor [22] were present. Then, we analyzed if a correlation existed between the observed symptom enhancement and changes in the accumulation of viral RNA in agroinfiltrated leaves at 5 dpi, and from young non-inoculated leaves at 12, 19, and 26 dpi. As shown in Figure 2B, the enhanced virus virulence observed for TRVp22 correlated with enhanced accumulation of viral RNA at 5, 12, and 19 dpi (analysis could not be performed at 26 dpi since TRV2p22-inoculated plants had died by this time).

In contrast to this, the expression of the suppressor p22 from a 16K suppressor deficient TRV mutant (TRV∆16Kp22 infections), in either agroinfiltrated patches or non-inoculated leaves, did not result in increased viral RNA accumulation, with levels always lower than that observed for wild type TRV-inoculated plants. In fact, by 19 and 26 dpi, the viral RNA accumulation in plants infected with TRV∆16Kp22, when p22 was present, were similar to the viral RNA levels observed for TRV∆16K infections, when neither 16K and p22 suppressors were present (Figure 2B). The differences in size clearly observed for genomic TRV RNA2 of the TRV∆16Kp22 constructs indicated that the ToCV p22 ORF cloned was retained in the viral progeny. Similar results were obtained in two independent experiments.

Taken together, these results indicate that the expression of the suppressor p22 from the heterologous vector TRV when the 16K suppressor is present enhances both symptomatology and viral RNA accumulation, but it cannot functionally complement a 16K defective virus at either the local and systemic level.

### 3.2. Enhanced Virulence with Heterologous Expression of the p22 Suppressor from a TRV Vector Is Not Linked to Faster Systemic Spread

**Figure 3:**
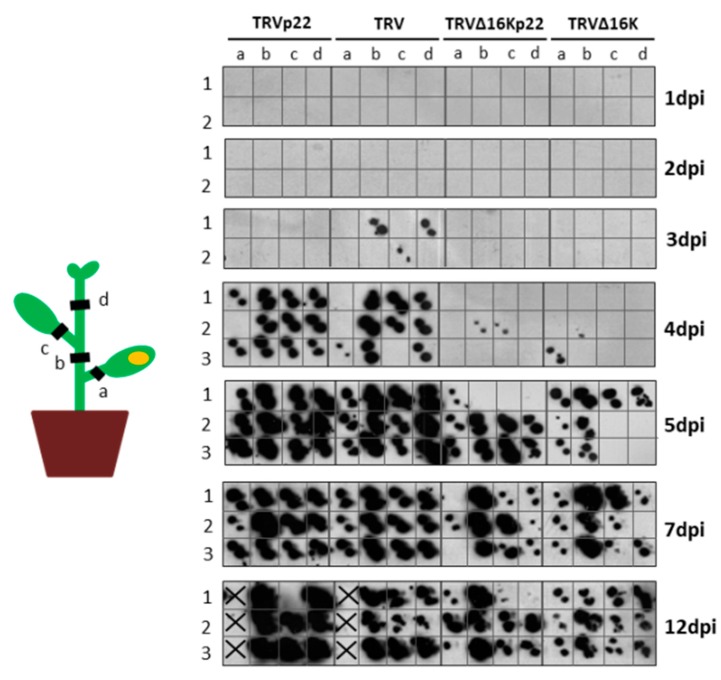
Analysis of the viral systemic spread in *Nicotiana benthamiana* plants inoculated with Tobacco rattle virus (TRV) expressing Tomato chlorosis virus p22 (TRVp22), TRV, TRV∆16Kp22, and TRV∆16K. Plant stem or leaf petiole tissue cross-sections were prepared from several plant locations (schematically represented at the left of the figure); (**a**) petiole of the agroinfiltrated leaf, (**b**) stem region between the agroinfiltrated leaf and the leaf above the agroinfiltrated one, (**c**) petiole of the leaf above the agroinfiltrated one, (**d**) youngest stem part of the plant. Groups of two or three plants were analyzed at each 1, 2, 3, 4, 5, 7, and 12 dpi. Hybridization was carried out with a probe specific to the 3′untranslatable region that is identical in RNA1 and RNA2 of TRV. X indicates that no cross-section is present.

We examined whether the rapid spread of the necrotic symptoms observed in plants infected with TRVp22 was linked to faster systemic spread of the virus. Tissue blot analysis was performed for cross-sections of non-inoculated tissues in four different locations of the plants (Figure 3, left) co-infiltrated with a mixture of TRV1 plus TRV2p22 or TRV2, and TRV1∆16K plus TRV2p22 or TRV2 at 1, 2, 3, 4, 5, 7, and 12 dpi. As shown in Figure 3, the first positive signal in non-inoculated parts of the plant was observed for plants inoculated with wild type TRV at 3 dpi. By 4 dpi, complete systemic spread was observed in plants inoculated with either TRVp22 and wild type TRV. By this time, however, the systemic spread was less evident in most TRV∆16Kp22 or TRV∆16K-inoculated plants. By 5 dpi, in almost all cases, the mutant or wild type viruses had spread from the inoculated to the young non-inoculated parts of the plant, although the systemic spread was more evident at 7 and 12 dpi. Similar results were obtained in two independent experiments.

These results show that the enhanced disease symptoms and virus accumulation in TRV2p22 infected plants do not seem to be linked to a faster spread of the chimeric virus across the plant. In addition to this, the presence of the suppressor p22 could not complement the absence of 16K in a defective TRV mutant virus.

### 3.3. Effect of the Heterologous Expression of the p22 Suppressor on PVX Virulence

**Figure 4:**
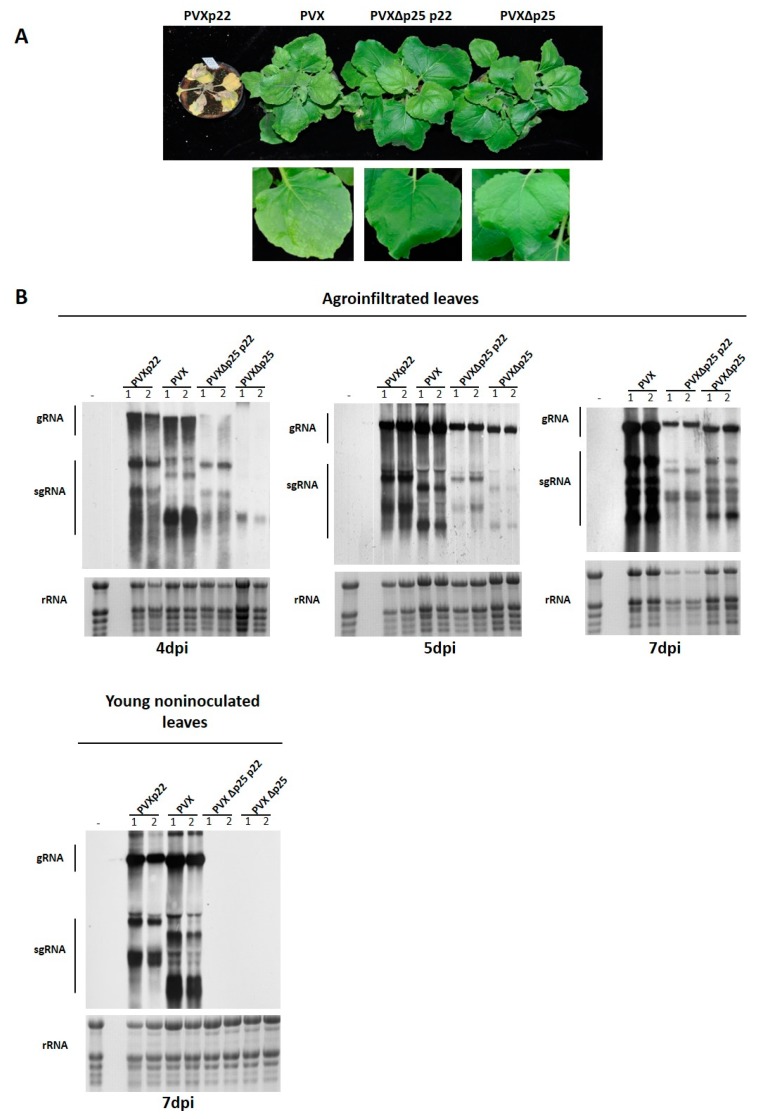
Analysis of *Nicotiana benthamiana* plants inoculated with Potato virus X (PVX) expressing Tomato chlorosis virus p22 (PVXp22), PVX, PVX∆P25p22, and PVX∆P25. (**A**) Symptoms on *N. benthamiana* plants following inoculation with PVXp22, PVX, PVX∆P25p22, and PVX∆P25 at 12 days post-inoculation. For PVX, PVX∆P25p22, and PVX∆P25 inoculations, a detail of a leaf systemically infected is shown; (**B**) Northern blot analysis of RNA extracted from agroinfiltrated patches at four, five, and seven days post-infiltration (dpi) and from non-inoculated young leaves at 7 dpi of plants agroinfiltrated with constructs PVXp22, PVX, PVX∆P25p22, and PVX∆P25. Hybridization was carried out with a probe specific to the 3′-end region of PVX. Positions of PVX genomics RNA (gRNA) and subgenomic RNAs (sgRNA) are indicated. Note that gRNA and sgRNA show slower electrophoretic mobility in PVXp22 and PVX∆P25p22 because of the insertion of the p22 gene sequence—is a healthy negative control. Ethidium bromide staining of rRNA was used as a loading control.

Although it was previously demonstrated that the expression of the suppressor protein p22 from a heterologous PVX vector enhanced the disease symptoms of infected plants [16], a detailed time-course analysis of viral accumulation was not conducted. In addition to this, as described above for TRV, a detailed analysis of the role of p22 in the context of a heterologous PVX infection was obtained by expressing p22 from the wild type PVX (PVXp22) and from a P25 suppressor deficient PVX mutant (PVX∆P25p22) (Figure 1B). Compared with PVX infection that resulted in mild mosaic symptoms in *N. benthamiana* plants at 7–8 dpi, plants inoculated with PVXp22 exhibited evident leaf curling in young non-inoculated leaves that rapidly progressed into generalized necrosis that led to plant death by 10–12 dpi (Figure 4A). In contrast, no symptoms were observed in plants infected with PVX∆P25p22 or PVX∆P25, in which neither p22 or a suppressor were present, respectively (Figure 4A). The necrotic symptoms observed progressed so quickly that a time course for the comparative analysis of virus accumulation could only be performed at 4, 5, and 7 dpi in agroinfiltrated leaves (although in the latter case, the necrosis of the patch did not allow for the extraction of RNA for agroinfiltrations with PVXp22), and at 7 dpi in non-inoculated leaves. By 7 dpi, although the non-inoculated leaves of PVXp22 infected plants already exhibited necrotic symptoms, it was still possible to extract total RNA to perform the analysis. As summarized in Figure 4B, analysis of agroinfiltrated patches at 4 and 5 dpi and young non-inoculated leaves at 7 dpi showed no obvious differences in the viral accumulation of PVXp22 and PVX, so it was not possible to link the enhanced symptoms observed with an increase in PVXp22 accumulation. In agroinfiltrated patches with PVX∆P25p22 or PVX∆P25, while at 4 dpi the viral accumulation of genomic RNA were delayed compared with PVXp22 and PVX agroinfiltrations (only subgenomic RNAs are clearly visible, showing a higher intensity in the case of the PVX∆P25p22 construct), at 5 and 7 dpi, similar but lower accumulation of both genomic and subgenomic RNAs were observed compared to those detected for PVXp22 and/or PVX. The slower electrophoretic mobility of genomic and subgenomic RNAs observed for the recombinant constructs PVXp22 and PVX∆P25p22, indicated that the p22 sequence was retained in the viral progeny. The absence of detection of PVX∆P25p22 or PVX∆P25 at 7 dpi in the young non-inoculated leaves of test plants indicates the strong constraints of the two P25 deleted mutant chimeras with regard to reaching distant parts of the plant. Similar results were obtained in two independent experiments.

Altogether, these results indicate that the expression of the suppressor p22 from the heterologous vector PVX when the P25 suppressor is present resulted in an accentuation of disease symptoms in inoculated plants that was not linked to an increase in viral RNA accumulation. The presence of p22 could not functionally complement a P25 defective virus at both the local and systemic level.

### 3.4. The Increased Virulence of the Recombinant Virus PVXp22 Is Not Linked to Faster Systemic Spread

**Figure 5:**
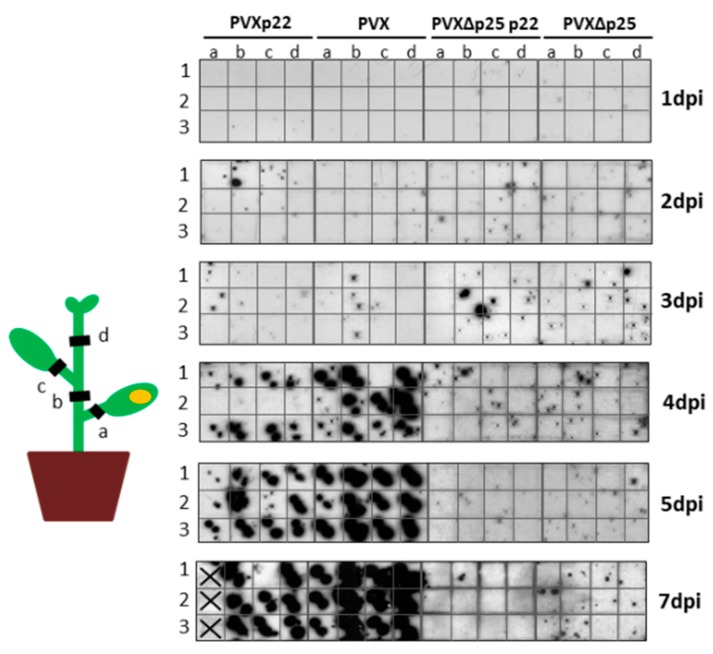
Analysis of the viral systemic spread in *Nicotiana benthamiana* plants inoculated with Potato virus X (PVX) expressing Tomato chlorosis virus p22 (PVXp22), PVX, PVX∆P25p22, and PVX∆P25. Cross sections of plant tissues in positions a, b, c, and d (schematically represented at the left of the figure) were done as described in Figure 3. Groups of three plants were analyzed at 1, 2, 3, 4, 5, and 7 dpi. Hybridization was carried out with a probe specific to the 3′untranslatable region of PVX. X indicates that no cross-section is present.

As described for TRV, we examined whether the rapid spread of the necrotic symptoms observed in plants infected with PVXp22 was linked to faster spread of the virus to non-inoculated parts of the plant by hybridization analysis of squash blots of stem or leaf petiole cross-sections of non-inoculated parts of the plant performed at 1, 2, 3, 4, 5, and 7 dpi (Figure 5). No differences were observed between PVXp22 and PVX indicating that the first clear positive signals of virus presence in the four sections of non-inoculated parts of the plants was observed at 4 dpi for both viruses. This positive signal was maintained in these two cases until 7 dpi, although the systemic necrosis shown by PVXp22-infected plants did not allow us to perform tissue blots of some cross-sections by 7 dpi. On the other hand, no positive signals in the non-inoculated parts of the plants were detected for PVX∆P25p22 or PVX∆P25 inoculations. Similar results were obtained from two independent experiments.

Taken together, these observations suggest that the heterologous expression of the suppressor p22 from a PVX vector does not result in faster spread of the virus throughout the plant and that p22 cannot functionally complement the suppressor defective virus PVX∆P25.

## 4. Discussion

Since RNA silencing suppressors have been linked to an increase in viral pathogenicity, we studied whether the p22 suppressor of the crinivirus ToCV, which exhibited a long-lasting suppressor activity in local assays in *N. benthamiana* [16], induced an enhancement in disease symptoms and/or viral accumulation when expressed from heterologous viruses. Although phloem limited viruses of the genus *Crinivirus,* and even ToCV, have been associated before to an enhancement of the symptomatology and viral accumulation when they are present in mixed infections [38,39,40,41,42] the approaches used to elucidate this effect have been different, not studying the specific region of the viral genome responsible. In our study, the role of ToCV p22 suppressor in heterologous viral infections was studied by expressing it from TRV and PVX viruses, and from their 16K and P25 suppressor deficient mutants, respectively.

The expression of ToCV p22 from TRV vector resulted into an enhancement of the pathogenicity and accumulation of the viral RNAs in plants. This enhancement, however, was not linked to a faster virus systemic spread. The fact of finding that the p22 expression increased the viral accumulation of TRV could be explained, at least in part, by the combined action of different suppressors on multiple steps of the RNA silencing pathway [3,43]. This interaction can contribute to a more effective way of counteracting an antiviral RNA silencing response in plants, which might then result in the observed higher viral accumulation. TRV 16K has been described as a relatively weak suppressor that effectively suppresses RNA silencing in the presence of low levels of dsRNA inducer [25]. Since its activity is overcome when a dsRNA inducer accumulates, the presence of ToCV p22 that can bind to long dsRNAs [17] could enhance the function of TRV 16K. The fact that the increased viral accumulation observed were not linked to faster systemic spread of TRVp22 could be due to the extra foreign sequence that may have affected TRV spread. Although the TRV vector has been described as very amenable to the insertion of heterologous sequences, as these sequences are not deleted during virus multiplication [19], we could speculate that the nature of the sequence expressed, the protein p22 could induce a plant response or alter the virus conformation in such a way that it could affect the systemic spread of this chimeric viral construct. A similar behavior has been observed for a TRV vector tagged with GFP to silence genes, where the phenotype of gene silencing in TRV-GFP infected *N. benthamiana* plants was delayed as compared with plants infected with the original TRV vector [44]. Although the presence of ToCV p22 resulted in the exacerbation of disease symptoms when the 16K TRV suppressor was present, it could not functionally complement a 16K defective suppressor deficient TRV mutant virus which induced a mild phenotype, not affecting the viral accumulation in either agroinfiltrated or non-inoculated leaves, or viral spread. In contrast to this, the heterologous expression of Cucumber mosaic virus (CMV, genus *Cucumovirus*, family *Bromoviridae*;) 2b, Pea early browning virus (PEBV, genus *Tobravirus,* family *Virgaviridae*) 12K, Soil-borne wheat mosaic virus (SBWMV, genus *Furovirus*, family *Virgaviridae*) 19K, and Barley stripe mosaic virus (BSMV, genus *Hordeivirus*, family *Virgaviridae)* γb suppressors from a 16K deficient mutant TRV virus were able to compensate the absence of the TRV 16K protein [28]. Although, as stated before, the mechanism of action of ToCV p22 could contribute to the TRV 16K suppressor activity, other factors are probably important for functional interchangeability. The fact that PEBV 12K, SBWMV 19K, BSMV γb and TRV 16K are all classified as cysteine-rich proteins (CRPs) could indicate that it is important to have both structural and functional similarity to be interchangeable. This conservation/complementation of action could be linked, for example, to a required subcellular distribution, necessary to accomplish a function. On the other hand, although the TRV1 encoded 29K protein has been described as a suppressor of RNA silencing in addition to 16K [22], our results in plants infected with the mutant recombinant virus TRV∆16Kp22 seemed to indicate that no interaction occurred between this protein and p22 when both suppressors were present simultaneously on a TRV background.

In the case of the heterologous expression of ToCV p22 from PVX, although we observed an enhancement of symptoms leading to plant death at 10–12 dpi, no differences in the viral RNA accumulation were observed in either agroinfiltrated or non-inoculated leaves of *N. benthamiana*. Similar results have been reported in *N. benthamiana* infected with PVX or PVX expressing the silencing suppressor HC-Pro either derived from PVY or TEV [3,8]. It was argued that the lack of an enhancement in PVX titers during this synergistic interaction in *N. benthamiana* could be due to the existence of a threshold above which viral accumulation cannot increase in this host, despite the inhibition of antiviral defenses by HC-Pro [8]. Therefore, since the enhanced pathogenicity associated with the heterologous expression of the suppressor p22 from PVX does not seem to be the consequence of a more efficient viral accumulation, other mechanisms must be involved. It is unlikely that the fast spread of the necrosis symptomatology observed in plants inoculated with PVXp22 could be due to a cytotoxic effect of p22, because in previous studies, high levels of p22 expression in agroinfiltrated patches was not linked to a necrotic phenotype [16]. Previous studies [45] indicate that the combination of PVX with different viral suppressors capable of inducing a systemic necrosis response in *N. benthamiana* do not have any effect in the PVX genomic RNA levels, but enhance and/or stabilize PVX subgenomic RNAs. In our case, we did not see a remarkable increase in the accumulation of subgenomic RNAs. However, at 4 dpi, the longest of the subgenomic RNAs show a higher intensity in the case of the recombinant viruses that express p22, even though these subgenomic RNAs are longer than in wild type PVX (Figure 4B). Thus, these authors propose that the systemic necrosis observed during PVX-associated synergism is a delayed systemic HR-like response triggered by P25 once it reaches a threshold level as a result of the action of the heterologous suppressor of silencing [44]. It has been suggested that *Nicotiana* spp. could carry a matching R gene that can specifically recognize P25 and mount an HR, where the suppressor activity of the P25 protein is necessary. Similar to what we observed for TRV, expression of p22 was unable to compensate for the impaired infection of a P25-suppressor defective mutant PVX. Although it can be argued that the frameshift mutation introduced in P25 would affect the RNA silencing suppression and movement functions, both necessary for PVX movement [35], this does not diminish the importance of the results obtained when p22 is expressed in this chimera. Thus, at the local level, the results obtained when p22 is expressed in a P25-suppressor defective mutant PVX show that, although ToCV p22 suppresses RNA silencing in local assays very efficiently [16], it is not capable of restoring the wild type viral accumulation of PVX when it is expressed by a P25-suppressor defective mutant PVX. It should also be highlighted that studies performed to determine if p22 could complement a P25-suppressor defective mutant PVX during systemic infection also have importance. Thus, although detailed studies have been performed to show that p22 functions as a suppressor of RNA silencing with a description of its mechanism of action [16,17,46], no other studies have been performed to assess if it is also involved in other points of the infection cycle, like virus movement. In fact, the existence of complex relationships has been reported between the suppression of RNA silencing and viral movement, as with existing suppressors whose movement phenotypes have been considered secondary manifestations of RNA silencing suppression activity [47,48,49,50]. In our case, the lack of movement complementation when ToCV p22 is expressed using a P25-suppressor defective mutant PVX impeded in viral movement indicates no existence of a potential plant cell-to-cell movement function for p22, at least when it is expressed by this heterologous viral vector. On the other hand, the expression of p22 by wild-type virus seemed to further limit the spreading ability of the virus (see Figure 5), probably due to the debilitating effect of the extra sequence.

In summary, the results presented here show that the combined action of non-related RNA silencing suppressors leads to an enhancement in disease severity, providing additional evidence for the existence of a link between viral suppression of RNA silencing and virulence.

## Supplementary Materials

The supplementary material is available online at www.mdpi.com/1999-4915/9/12/358/s1.
