# Supplementary material for: The Heterologous Expression of the p22 RNA Silencing Suppressor of the Crinivirus Tomato Chlorosis Virus from Tobacco Rattle Virus and Potato Virus X Enhances Disease Severity but Does Not Complement Suppressor-Defective Mutant Viruses
Source: Viruses. 2017 Nov 24;9(12):358. doi: 10.3390/v9120358 (PMC5744133; doi:10.3390/v9120358)
Supplement: Supplementary file 1 [file viruses-09-00358-s001.docx]

| **Supplementary Table S1** | |  |  |  |
| --- | --- | --- | --- | --- |
| **Sequences of primers used in this work** | | | | |
| **Genome** | **Primer** |  | **Primer sequence (5' - 3')** | **Primer position** |
| TRV | MA 1281 | (FW) | TACTCAAGGGTTGTGTGAAT***TAA***GTCACTGTTCTTGGTCAC | 6121 |
| TRV | MA 1282 | (RV) | GTGACCAAGAACAGTGAC***TTA***ATTCACACAACCCTTGAGTA | 6161 |
| PVX | MA 1621 | (FW) | CTTTAGATTCA***TGA***CCT***TAG***GTAGTACATGCAGTAGCC | 5543 |
| PVX | MA 1622 | (RV) | GGCTACTGCATGTACTAC***CTA***AGG***TCA***TGAATCTAAAG | 5580 |
| ToCV | MA 1287 | (FW) | ACCGAATTCATGGATCTCACTGGTTGTTTGC | 7662 |
| ToCV | MA 1288 | (RV) | TAACTCGAGTTATATATCACTCCCAAAGAAAAAC | 8243 |
| **Table S1:** The underlined sequences are sites restriction endonucleases introduced into primers for cloning (*Eco* RI in MA 1287 and *Xho* I in MA 1288). In bold and italic sequences indicate the stop codons introduced into primers. Primer positions indicate the positions in: RNA1-TRV genome of Ppk20 isolate with GenBank access number AF166084, pgR107 vector with GenBank access number AY297842 and RNA1-ToCV genome of AT 80/99 isolate with GenBank access number DQ983480. FW and RV indicate forward and reverse primers sense. | | | | |
